# pVAXhsp65 Vaccination Primes for High IL-10 Production and Decreases Experimental Encephalomyelitis Severity

**DOI:** 10.1155/2017/6257958

**Published:** 2017-02-21

**Authors:** Sofia Fernanda Gonçalves Zorzella-Pezavento, Fernanda Chiuso-Minicucci, Thais Graziela Donegá França, Larissa Lumi Watanabe Ishikawa, Larissa Camargo da Rosa, Priscila Maria Colavite, Bianca Balbino, Camila Marques, Maura Rosane Valerio Ikoma, Ana Paula Masson, Célio Lopes Silva, Alexandrina Sartori

**Affiliations:** ^1^Department of Microbiology and Immunology, Institute of Biosciences of Botucatu, Universidade Estadual Paulista (UNESP), Botucatu, SP, Brazil; ^2^Flow Cytometry Laboratory, Amaral Carvalho Foundation, Jaú, SP, Brazil; ^3^Department of Biochemistry and Immunology, University of São Paulo (USP), Ribeirão Preto, SP, Brazil

## Abstract

Experimental autoimmune encephalomyelitis (EAE) is a demyelinating pathology of the central nervous system (CNS) used as a model to study multiple sclerosis immunopathology. EAE has also been extensively employed to evaluate potentially therapeutic schemes. Considering the presence of an immune response directed to heat shock proteins (hsps) in autoimmune diseases and the immunoregulatory potential of these molecules, we evaluated the effect of a previous immunization with a genetic vaccine containing the mycobacterial hsp65 gene on EAE development. C57BL/6 mice were immunized with 4 pVAXhsp65 doses and 14 days later were submitted to EAE induction by immunization with myelin oligodendrocyte glycoprotein (MOG_35–55_) emulsified in Complete Freund's Adjuvant. Vaccinated mice presented significant lower clinical scores and lost less body weight. MOG_35–55_ immunization also determined less inflammation in lumbar spinal cord but did not change CD4+CD25+Foxp3+ T cells frequency in spleen and CNS. Infiltrating cells from the CNS stimulated with rhsp65 produced significantly higher levels of IL-10. These results suggest that the ability of pVAXhsp65 vaccination to control EAE development is associated with IL-10 induction.

## 1. Introduction

Multiple sclerosis (MS) is a progressive inflammatory disease that damages the brain and the spinal cord. A plethora of reports support the view that it is mediated by autoreactive T cells specific for myelin antigens [1, 2]. Once they had been activated in the periphery, these self-specific T cells cross the blood-brain barrier and destroy the myelin sheets and axons from central neurons [3–5]. This demyelination is responsible for signal conduction slowing or even signal blockage [6]. Currently available therapies for MS are primarily focused in minimizing the progression of disability and reducing the number of relapses. The standard first-line immunomodulatory therapies include IFN-*β* and glatiramer acetate [7, 8]. Other drugs as natalizumab and fingolimod are also effective in lowering recurrence rates and slowing disease progression [9]. Experimental autoimmune encephalomyelitis (EAE) is an induced demyelinating pathology of the central nervous system (CNS) that is commonly used as a model to investigate this disease. EAE is triggered in susceptible mouse and rat strains by immunization with myelin proteolipid protein, myelin basic protein, or myelin oligodendrocyte glycoprotein (MOG_35–55_) emulsified in Complete Freund's Adjuvant (CFA) [10–12]. These models and also human studies indicate that many cell subsets as Th1, Th17, Tc, and T*γδ* contribute to damage of the CNS [1, 13]. More recently, subsets of T cells endowed with immunoregulatory ability were characterized and their contribution to restrain self-reactivity is being established. Regulatory T cells (Treg cells) encompass both natural and inducible (adaptative) cell types. Natural Treg cells are identified by high CD25 expression and intracellular presence of the forkhead box P3 (Foxp3) transcription factor, which is required for directing regulatory function [14]. These cells were originally identified by their ability to establish tolerance to self-antigens [15]. Natural Treg cells develop in the thymus after expression of Foxp3 at a relatively late stage of thymopoiesis [16]. Adaptive Treg cells including Tr1, Th3, and various CD8+ Treg cells subsets are triggered by stimulation of naïve T cells by their cognate antigens (self or nonself) in the periphery. Suppressive cytokines as IL-10 and TGF-*β* contribute to both induction of these Treg cells and also stimulation of their suppressive function. Tr1 cells exert their suppressive function primarily through IL-10 secretion [17] whereas Th3 cells act mainly through TGF-*β* release [18].

Even though current therapies for MS are limiting the impact of this neurodegenerative disorder, they can cause severe side effects as cutaneous lesions [19], depression, thyroid dysfunction, cardiotoxicity, liver enzymes abnormalities, and increased susceptibility to infections [20]. The production of anti-drug antibodies has also been described [21]. Novel disease modifying therapies are being tested as monoclonal antibodies, chimeric molecules, and oral therapies [22, 23]. Prophylactic and therapeutic approaches based on Treg cells induction would be highly useful. Even though antigen-specific Treg cells would be preferentially indicated, other Treg cells subsets are being examined. Heat shock proteins (hsps), for example, are being considered as potential targets for the treatment of inflammatory diseases due to their increased expression in inflammatory foci. This possibility is supported by convincing evidence that hsps can induce immunoregulatory T cell responses [24–27]. The immunomodulatory ability of the mycobacterial hsp65 in autoimmune diseases has been demonstrated by us and other authors in arthritis [28, 29], diabetes [30–32], and atherosclerosis [27, 33].

More recently, we also tested the immunomodulatory potential of pVAXhsp65 (a genetic vaccine containing the hsp65 mycobacterial gene) in EAE. Initially it was applied therapeutically, that is, after disease induction. Even though this approach was able to significantly downmodulate the peripheral production of encephalitogenic cytokines, it was not capable of reducing disease severity [34]. Considering that memory and naïve T cells could present a differential susceptibility to regulation [35], we supposed that naïve T cells, specific for MOG_35–55_ and present in mice not yet submitted to EAE induction, could be more responsive to regulation by hsp65. We therefore tested the prophylactic potential of pVAXhsp65 on EAE development.

## 2. Materials and Methods

### 2.1. Experimental Procedure

A preliminary experiment was done to choose the immunization protocol with the highest immunoregulatory potential. Mice were injected with 2, 3, or 4 doses of pVAXhsp65 with 14 days being the interval between doses. The animals were euthanized 14 days after the last dose and IFN-*γ* and IL-10 were quantified in spleen cell cultures stimulated with recombinant hsp65 (rhsp65). Mice injected with saline were used as a control group. To evaluate the prophylactic effect of pVAXhsp65 on EAE development, mice were immunized with 4 pVAXhsp65 doses and 14 days after last dose they were submitted to EAE induction. Disease development was evaluated by clinical follow-up (clinical score and weight variation) and also by histopathological analysis of the CNS. The immunoregulatory potential of pVAXhsp65 vaccination was checked by the profile of cytokine production by spleen and CNS infiltrating cells stimulated with MOG_35–55_ or rhsp65 and also by the presence of Foxp3+ regulatory T cells in these two organs. Mice injected with saline (control) or with the empty vector (pVAX) were used as control groups.

### 2.2. Animals

Female C57BL/6 mice (4–6 weeks old) were purchased from CEMIB (UNICAMP, São Paulo, SP, Brazil). The animals were fed with sterilized food and water ad libitum and were manipulated in accordance with the ethical guidelines adopted by the Brazilian College of Animal Experimentation. Animal experiments were conducted with the approval of the Ethics Committee for Animal Experimentation, Medical School, Universidade Estadual Paulista.

### 2.3. DNA Vaccine Encoding hsp65 Assembly

The vaccine pVAXhsp65 was constructed from the pVAX vector (Invitrogen, Carlsbad, CA, USA). This plasmid was digested with BamHI and NotI (Gibco BRL, Gaithersburg, MD, USA) and then the* M. leprae* hsp65 gene and the CMV intron A were inserted. DH5*α E. coli* transformed with pVAX or pVAXhsp65 were cultured in LB liquid medium (Gibco BRL, Gaithersburg, MD, USA) containing kanamycin (100 *μ*g/ml). The plasmids were purified using the Concert High Purity Maxiprep System (Gibco BRL, Gaithersburg, MD, USA). Plasmid concentrations were determined by spectrophotometry at *λ* = 260 and 280 nm by using the Gene Quant II apparatus (Pharmacia Biotech, Buckinghamshire, UK).

### 2.4. Recombinant hsp65 Protein (rhsp65)

The rhsp65 was obtained from* Escherichia coli* ER2566 previously transformed with the hsp65 gene from* M. leprae*. The transfected* E. coli* was cultured in LB containing ampicillin (100 *µ*g/*µ*l) and the bacterial growth was monitored by spectrophotometry at 600 nm. When the optic density reached a value of 0.6, the culture was induced with isopropylthiogalactoside 0.1 M (Gibco BRL, Gaithersburg, MD, USA) and incubated at 30°C under agitation for 4 h. Details of rhsp65 production and purification can be found at dos Santos et al., 2010 [36].

### 2.5. Vaccination with pVAXhsp65

Mice were injected with 4 doses of pVAXhsp65 (100 *μ*g/100 *μ*l) by intramuscular route (quadriceps muscle). Fourteen days after the last dose the animals were submitted to EAE induction.

### 2.6. EAE Induction

MOG_35–55_ peptide (MEVGWYRSPFSRVVHLYRNGK) was synthesized by Proteimax, São Paulo, Brazil. EAE was induced as previously reported [37]. Briefly, mice were immunized with 150 *μ*g of MOG_35–55_ emulsified in CFA containing 400 *μ*g of BCG. Two doses of 200 ng of* Bordetella pertussis* toxin (Sigma Aldrich, St. Louis, MO, USA) were administered by intraperitoneal route. Animals were daily checked and disease intensity was recorded as follows: (0) no symptoms, (1) limp tail, (2) hind legs weakness, (3) partially paralyzed hind legs, (4) complete hind leg paralysis, and (5) complete paralysis/death.

### 2.7. CNS Infiltrating Mononuclear Cells Isolation

Mononuclear cells infiltrated in the CNS were obtained as previously described by Mimura et al., 2016 [38]. Briefly, sedated (ketamine/xylazine) mice were perfused with saline solution and then brain and the whole spinal cord were collected, macerated, resuspended in RPMI medium (Sigma Aldrich) supplemented with 2.5% collagenase D (Roche Applied Science) and incubated at 37°C, 5% CO_2_ incubator for 45 min. Cells were then resuspended in percoll (GE Healthcare) 37% and gently laid over percoll 70%. After centrifugation at 950 ×g for 20 min the ring containing mononuclear cells was collected. Cellular suspension from percoll interface was then resuspended in supplemented RPMI medium (1% gentamicin, 2% glutamine, 1% sodium pyruvate, 1% nonessential amino acids, and 10% of fetal calf serum).

### 2.8. Spleen and CNS Cell Culture Conditions

Spleen and mononuclear cells isolated from the CNS were adjusted to 5 × 10^6^ cells/ml and 2 × 10^5^ cells/ml, respectively, and cultured in supplemented RPMI medium. Spleen cells were restimulated in vitro with MOG_35–55_ (20 *μ*g/ml) or rhsp65 (10 *μ*g/ml), while CNS-isolated cells were stimulated with MOG_35–55_ (50 *μ*g/ml) and rhsp65 (10 *μ*g/ml). IFN-*γ*, IL-10, IL-6, IL-17, and TNF-*α* levels were assessed in culture supernatants by ELISA (Becton Dickinson, San Jose, CA, USA, and R&D Systems, Minneapolis, MN, USA) according to the manufacturer's instructions.

### 2.9. Proportion of CD4+CD25+Foxp3+ T Cells

Spleen cells were collected and the red blood cells were lysed with Hank's buffer containing NH_4_Cl. Spleen and CNS infiltrating cells were adjusted to 2.5 × 10^6^ cells/100 *μ*l and then incubated 0.5 *µ*g of FITC labeled anti-mouse CD4 (clone GK1.5) and 0.25 *μ*g of APC labeled anti-mouse CD25 (clone PC61.5) at room temperature during 20 min. A staining for Foxp3 was performed using the anti-mouse/rat Foxp3 Staining Set (eBioscience, San Diego, CA, USA) according to the manufacturer's instructions. The cells were analyzed by flow cytometry using the FACSCanto II (Becton Dickinson, San Jose, CA, USA) and FACSDiva software (Becton Dickinson, San Jose, CA, USA) at Amaral Carvalho Foundation (Jaú, São Paulo, Brazil).

### 2.10. Inflammatory Infiltration in the CNS

The histological analysis was performed in the CNS at the 30th day after EAE induction. Lumbar spinal cord samples were removed and fixed in 10% neutral buffered formalin. Paraffin slides with 5 *μ*m were stained with hematoxylin and eosin (H&E) and analyzed with a Nikon microscope. A semiquantitative analysis of CNS inflammation was performed according to the following criteria: (0) no infiltrates; (1) partial meningeal infiltration; (2) pronounced meningeal infiltration; and (3) pronounced meningeal and some parenchymal infiltration as already adopted by us and other authors [39, 40]. This evaluation was done with a Nikon microscope by analyzing two distinct areas in the samples of each animal.

### 2.11. Statistical Analysis

Data were expressed as mean ± SE. Comparisons between groups were made by one-way ANOVA with post hoc Holm-Sidak test for parameters with normal distribution and by Kruskal-Wallis followed by a post hoc Dunn's test for parameters with nonnormal distribution. Significance level was *p* < 0.05. Statistical analysis was accomplished with SigmaStat for Windows v 3.5 (Systat Software Inc).

## 3. Results

### 3.1. Immune Response Induced by pVAXhsp65 Immunization

**Figure 1:**
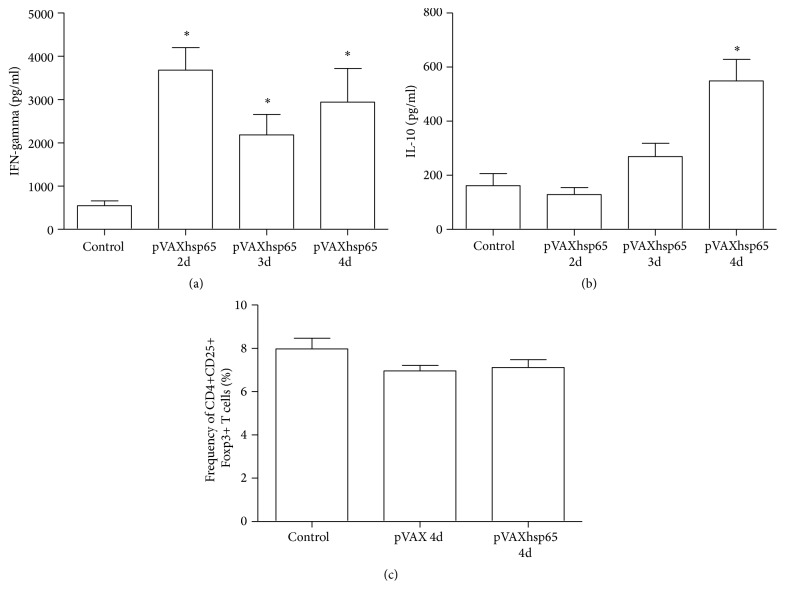
*Immune response induced by pVAXhsp65 vaccination*. C57BL/6 mice were immunized with 2, 3, or 4 pVAXhsp65 doses with 14 days' interval. IFN-*γ* (a) and IL-10 (b) production were assessed in spleen cells cultures restimulated in vitro with rhsp65. The percentage of CD4+CD25+Foxp3+ T cells was evaluated in the total number of spleen cells 14 days after the last dose of pVAXhsp65 (c). Data were presented by mean ± SE of 6 mice and are representative of two independent experiments. *∗* represents the difference between immunized and control group. *p* < 0.05.

Immunization with 2, 3, or 4 pVAXhsp65 doses determined the production of similar amounts of IFN-*γ* (Figure 1(a)) by spleen cells stimulated with rhsp65. However, only 4 pVAXhsp65 doses triggered a significant IL-10 production (Figure 1(b)) by these cells. These high IL-10 levels were not, however, associated with a higher frequency of CD4+CD25+Foxp3+ T cells in the spleen. The proportion of these cells, evaluated 14 days after DNA immunization, was similar in immunized, injected with vector or noninjected experimental groups (Figure 1(c)).

### 3.2. Decreased EAE Severity in Mice Previously Immunized with pVAXhsp65

**Figure 2:**
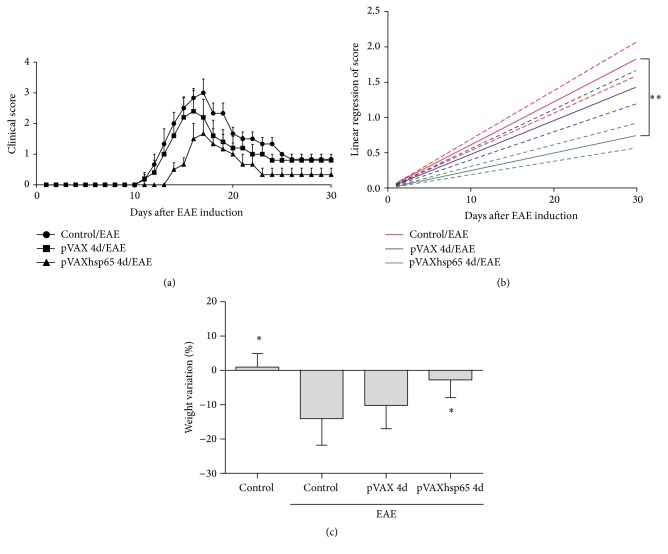
*Effect of previous vaccination with pVAXhsp65 on EAE development*. C57BL/6 mice were immunized with 4 pVAXhsp65 doses and then submitted to EAE induction. Kinetics of clinical scores (a), linear regression analysis of clinical scores (b), and body weight variation (c). Data were presented by mean ± SE of 6 mice and are representative of three independent experiments. *∗* represents the difference between immunized and control group with EAE. ^*∗*^*p* < 0.05 and ^*∗∗*^*p* < 0.001.

Previous immunization with pVAXhsp65 significantly reduced EAE symptoms. By the 17th day of the disease, when the paralysis achieved its maximum level in the EAE control group (clinical score = 3), the average score in the previously immunized group was 1.5. Previous vaccination also delayed disease onset and determined lower clinical scores during the chronic disease phase (Figure 2(a)). The statistical significance of clinical improvement was determined by linear regression analysis (Figure 2(b)). Besides, mice previously immunized with pVAXhsp65 lost significantly less weight than the EAE control group (Figure 2(c)).

### 3.3. CNS Inflammation in Mice Previously Immunized with pVAXhsp65

**Figure 3:**
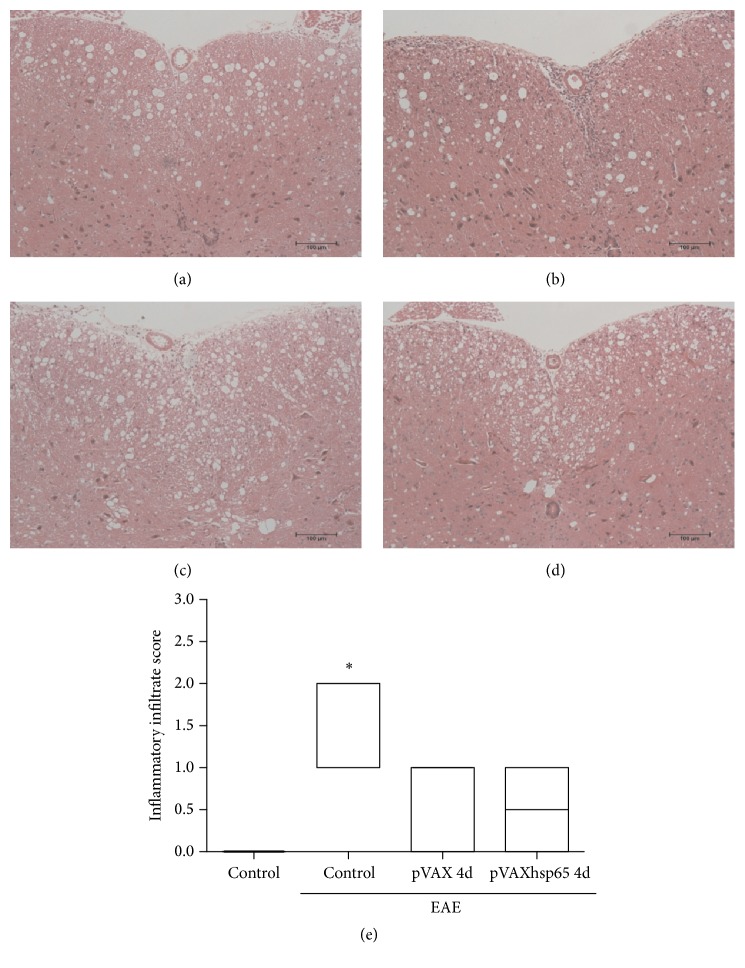
*Effect of previous immunization with pVAXhsp65 on CNS inflammation*. C57BL/6 mice were immunized with 4 pVAXhsp65 doses and then submitted to EAE induction. Lumbar spinal cord inflammatory infiltrates in control mice without EAE (a), control mice with EAE (b), and mice immunized with 4 pVAX (c) or pVAXhsp65 doses (d) were evaluated 30 days after EAE induction. A semiquantitative analysis was used to assess the inflammatory infiltration (e) during the chronic disease phase. Micrographs are representative of 6 animals/group. Data in panel (e) were presented by mean ± SE of 6 mice. *∗* represents the difference between immunized and control group with EAE. *p* < 0.05.

Typical lesions mainly characterized by mononuclear cell infiltration were observed in the meningeal areas of the CNS in mice with EAE (nonimmunized) during the chronic period of the disease. Semiquantitative microscopic analysis indicated that previous DNA immunization reduced the magnitude of inflammation in the lumbar spinal cord samples in comparison to nonimmunized animals as illustrated in Figure 3.

### 3.4. Peripheral Immune Response in Mice Previously Immunized with pVAXhsp65

**Figure 4:**
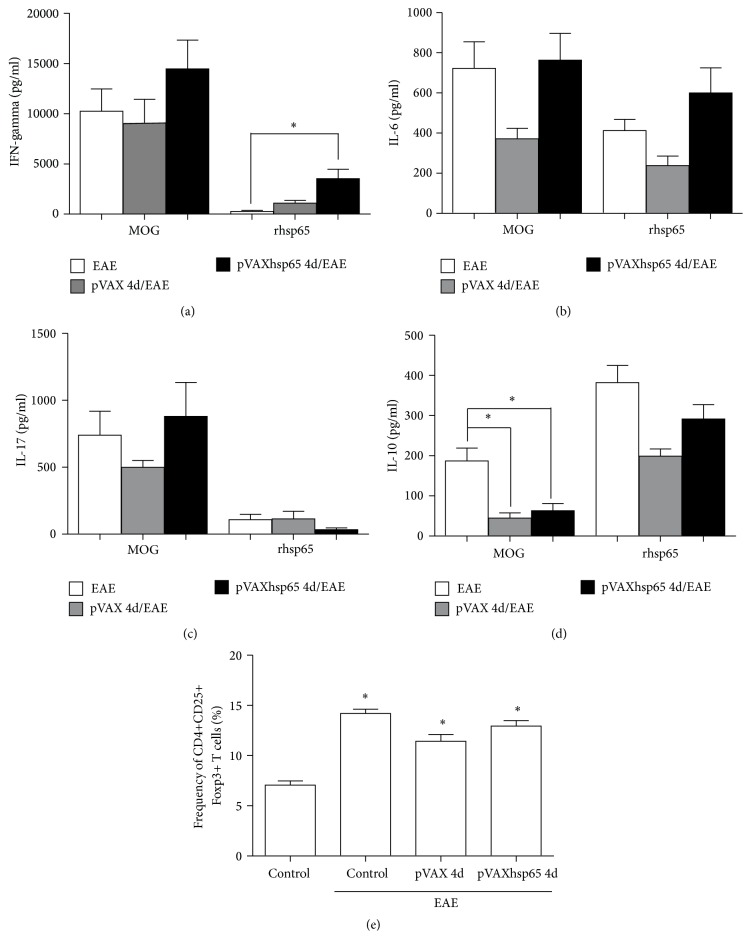
*Effect of previous vaccination with pVAXhsp65 on peripheral immune response*. C57BL/6 mice were immunized with 4 pVAXhsp65 doses and then submitted to EAE induction. Cytokine production was assessed 30 days after EAE induction. IFN-*γ* (a), IL-6 (b), IL-17 (c), and IL-10 (d) production were assayed in spleen cell cultures restimulated in vitro with MOG_35–55_ or rhsp65. The percentage of CD4+CD25+Foxp3+ T cells was evaluated in the total number of spleen cells 30 days after EAE induction (e). Data were presented by mean ± SE of 6 mice and are representative of two independent experiments. *∗* represents the difference between DNA injected groups and control group with EAE. *p* < 0.05.

Cytokine production by cultures from different experimental groups was compared 30 days after EAE induction. IFN-*γ* (Figure 4(a)), IL-6 (Figure 4(b)), and IL-17 (Figure 4(c)) produced by spleen cells from EAE, pVAX/EAE, and pVAXhsp65/EAE groups stimulated with MOG_35–55_ reached similar levels, whereas IL-10 production was similarly downmodulated in vector and vaccine previously injected groups (Figure 4(d)). A distinct cytokine profile was observed in cultures stimulated with rhsp65. In this case, only IFN-*γ* levels (Figure 4(a)) were significantly higher in the group previously vaccinated with pVAXhsp65. Levels of IL-6 (Figure 4(b)), IL-17 (Figure 4(c)), and IL-10 (Figure 4(d)) were similar in EAE, pVAX/EAE, and pVAXhsp65/EAE groups. EAE development was already associated with high levels of Foxp3+ regulatory T cells in peripheral lymphoid organs and pVAXhsp65 immunization did not augment the proportion of these regulatory T cells in the spleen (Figure 4(e)) when compared with the control groups (EAE and pVAX/EAE).

### 3.5. Immune Response at the CNS in Mice Previously Immunized with pVAXhsp65

**Figure 5:**
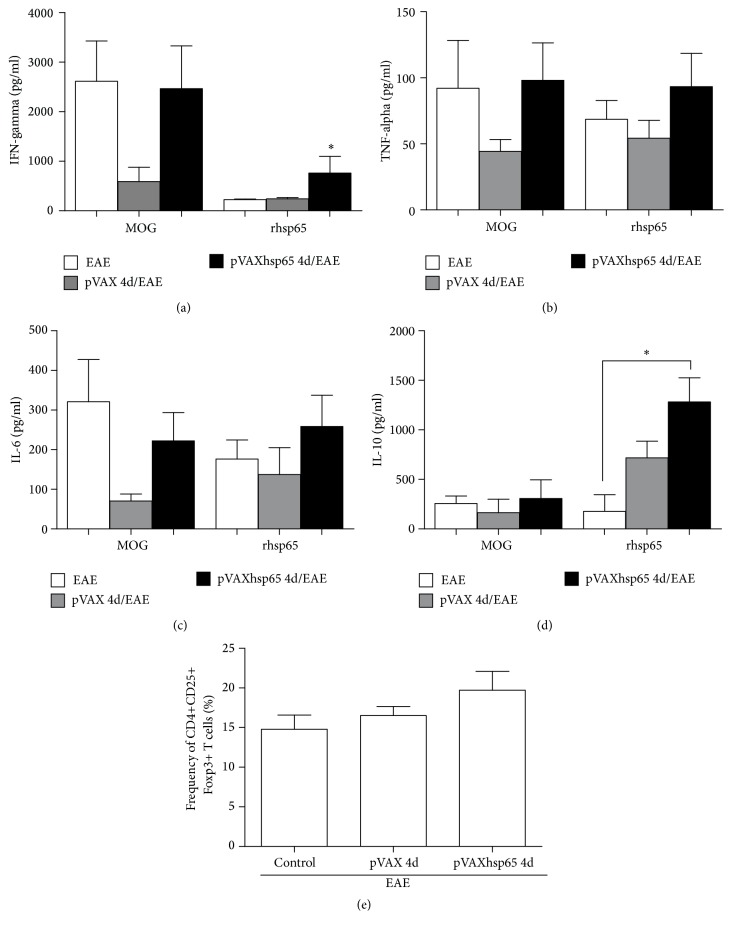
*Effect of previous vaccination with pVAXhsp65 at the CNS*. C57BL/6 mice were immunized with 4 pVAXhsp65 doses and then submitted to EAE induction. Cytokine production was assessed 30 days after EAE induction. IFN-*γ* (a), TNF-*α* (b), IL-6 (c), and IL-10 (d) production were assayed in CNS infiltrating cells cultures restimulated in vitro with MOG_35–55_ or rhsp65. The percentage of CD4+CD25+Foxp3+ T cells was evaluated in the total number of mononuclear cells from CNS 30 days after EAE (e). Data were presented by mean ± SE of 5 mice. *∗* represents the difference between immunized and control group with EAE. *p* < 0.05.

A significant production of all checked cytokines was detected in cultures from CNS infiltrating cells. The levels of IFN-*γ* (Figure 5(a)), TNF-*α* (Figure 5(b)), IL-6 (Figure 5(c)), and IL-10 (Figure 5(d)) were similarly elevated in the three experimental groups when the cells were stimulated with MOG_35–55_. The amounts of TNF-*α* (Figure 5(b)) and IL-6 (Figure 5(c)) were also comparable in cultures from these three groups stimulated with rhsp65. However, stimulation with rhsp65 triggered a significantly higher production of IFN-*γ* (Figure 5(a)) and IL-10 (Figure 5(d)) in mice that were previously immunized with pVAXhsp65. Similarly to the peripheral findings, pVAXhsp65 immunization also did not increase the frequency of CD4+CD25+Foxp3+ T cells in the CNS (Figure 5(e)).

## 4. Discussion

EAE is a widely employed model to understand MS pathogenesis and also to search for prophylaxis and new therapeutic measures towards this pathology. In this work, we found that a genetic construction containing the mycobacterial hsp65 gene is endowed with prophylactic application against EAE development. C57BL/6 mice were initially immunized with variable pVAXhsp65 doses to choose the potentially protective schedule. Only the 4-dose scheme was able to prime these animals for a higher IL-10 production. As IL-10 producer cells have been described as being induced by rhsp65 and also able to downmodulate autoimmune conditions [31], this vaccination procedure was chosen to investigate the prophylactic potential of this vaccine in EAE. C57BL/6 mice were then immunized with 4 pVAXhsp65 doses and then submitted to EAE induction. The positive control group, that is, only subjected to EAE induction, developed the classical signs of EAE as accentuated weight loss and clinical paralysis. These results were expected and very similar to what has been described by us and other authors that used the encephalomyelitis model induced by MOG_35–55_ immunization [41–43]. Previous vaccination with pVAXhsp65 clearly modified disease development. These mice lost less body weight and also showed lower clinical scores. The onset of clinical signs was likewise delayed in this experimental group. This protective effect was also associated with decreased inflammation at the lumbar spinal cord suggesting reduced migration of peripheral encephalitogenic T cells to the CNS.

Cytokine production by spleen cells stimulated with MOG_35–55_ highly suggested that the pVAXhsp65 is not working by decreasing the peripheral proinflammatory specific immune response, as IFN-*γ*, IL-17, and TNF-*α* levels were similar in all experimental groups. Intriguingly, IL-10 levels in these cultures were significantly downregulated in DNA injected mice. When these splenic cells were restimulated with rhsp65, the expected high IFN-*γ* and IL-10 levels previously observed in pVAXhsp65 immunized mice were not observed. As IL-10 has been described as one of the most effective anti-inflammatory cytokines in EAE and MS [44], we reasoned that IL-10 producer cells, specific for MOG_35–55_ or hsp65, had migrated to the CNS and partially controlled inflammation. This possibility was tested by stimulating mononuclear cells eluted from CNS with MOG_35–55_ and rhsp65. Mice previously immunized with pVAXhsp65 produced significantly higher levels of IFN-*γ* and IL-10 in response to rhsp65, but not MOG_35–55_, after in vitro restimulation. As nonstimulated cell cultures did not produce cytokines (data not shown), we hypothesized that these were hsp65 specific cells. Vaccination also did not alter the frequency of Foxp3+ Treg cells in the periphery or in the CNS, suggesting that the classical CD4+CD25+Foxp3+ Treg cells are not responsible for IL-10 production. It is possible therefore that other cell types that do not express Foxp3 are the source of this anti-inflammatory cytokine [45].

As the protocol that we used to isolate CNS cells was based on centrifugation over discontinuous percoll gradients, a variety of cells could be present in these CNS cell cultures as neuronal cells, astrocytes, oligodendrocytes, microglial cells, and infiltrating leukocytes, as described by Pino and Cardona, 2011 [46]. Then, distinct specific or even nonspecific cells could be the source of this regulatory cytokine. Various cell types as macrophages, dendritic cells, Tr1, and B regulatory cells [47, 48] are described as being able to produce IL-10 and to contribute to EAE and MS recovery. Many reports have also highlighted the role of hsp60/65 in activation of B cells [49], T cells [50], Treg cells [51], and maturation of dendritic cells [52]. Specially concerning hsp65 formulated as a DNA vaccine, Fontoura et al., 2015 [53], described that DNAhsp65 immunization of C57BL/6 mice induced a subtype of IL-10 producing B cell able to reduce the production of proinflammatory cytokine mRNAs in the spleen. Hsp65 was also able to attenuate the development of airway hyperresponsiveness and inflammation in BALB/c mice through modulation of dendritic cell function [54]. These findings endorse the possibility that hsp65 responsive cells able to produce IL-10 are present in the CNS and could mediate the protective effect observed in this work.

The vector also triggered a protective effect even though it was discrete comparing to the one elicited by the vaccine. This finding suggests that the immunoregulatory ability of pVAXhsp65 partially depends upon the plasmid vector itself. In this regard, CpG motifs in the plasmid vector could trigger an anti-inflammatory immune response. This possibility is supported by some literature reports. Quintana et al., 2000 [55], showed that injection of empty plasmid DNA or CpG oligonucleotides inhibited diabetes in NOD mice due to a shift to Th2 profile. More recently, it has been demonstrated that CpG-DNA sequences were capable of inducing a Th2 response in human endothelial cells through the inhibition of proinflammatory cytokines and enhanced IL-10 expression [56].

A similar anti-inflammatory effect of this genetic vaccine was previously described by our research group in arthritis [28] and diabetes [30, 31, 57] experimental models. The potential of pVAXhsp65 as a prophylactic vaccine against diabetes was also established by us in both homologous and heterologous prime-boost strategies. NOD mice were clinically protected by immunization with pVAXhsp65. The vector also determined immunomodulation but its protective effect against insulitis was very discrete. Interestingly, protection coincided with the influx of CD25+ cells and increased staining for IL-10 in the islets [30]. We also demonstrated that the combination of pVAXhsp65 with BCG, in a heterologous prime-boost protocol, was highly effective to prevent diabetes in NOD mice [57]. In addition, we observed that this vaccine decreased lumbar inflammation and downmodulated peripheral IL-10 production in an EAE rat model [12] similarly to the findings showed in the present investigation. Again, the empty plasmid prompted a similar but less pronounced effect. This protective effect of hsp65 in distinct inflammatory conditions is highly supported by findings of many other authors [58–61].

Concerning EAE development, the immunoregulatory effect of hsp65 has also been demonstrated by employing distinct formulations containing this heat shock protein. For example, oral administration of a recombinant* Lactococcus lactis* strain that produces hsp65 prevented the development of EAE in C57BL/6 mice. This protection was confirmed by the reduced inflammatory cell infiltrate and absence of injury signs in the spinal cord [62]. Also recently, Billetta et al., 2012 [63], found that intranasal treatment of EAE with the peptide RatP2 derived from hsp60 determined a significant clinical improvement which was superior to therapy with glatiramer acetate.

## 5. Conclusion

Previous vaccination with pVAXhsp65 was able to reduce EAE clinical manifestations and also triggered higher IL-10 production at the CNS. These findings reinforce the potential of hsp65 to be explored as an adjuvant therapy in this and other autoimmune pathologies.
